# Monocyte cluster identification during the early stage of fracture healing in an ovariectomized mouse model

**DOI:** 10.3389/fmed.2026.1803265

**Published:** 2026-04-08

**Authors:** Bo Zeng, Wei Wu, Ziyu Huang, Hao Li, Remila Aimaiti, Yongjian Zhao, Yide Fang, Liuqing Jing, Jinhai Xu, Jing Wang, Wen Mo, Qi Shi, Yongjun Wang, Yingjie Deng, Sheng Lu, Bing Shu

**Affiliations:** 1 Longhua Hospital, Shanghai University of Traditional Chinese Medicine, Shanghai, China; 2 Spine Institute, Shanghai Academy of Traditional Chinese Medicine, Shanghai, China; 3 Key Laboratory, Ministry of Education of China, Shanghai, China; 4 Xinjiang Medical University, Urumqi, China; 5 Affiliated Hospital of Traditional Chinese Medicine, Xinjiang Medical University, Urumqi, China; 6 Hospital of Traditional Chinese Medicine, Urumqi, China; 7 Academy of Traditional Chinese Medicine, Urumqi, China

**Keywords:** inflammation, monocytes, osteoporotic fracture, osteoporotic, single-cell RNA sequencing

## Abstract

**Background:**

Monocytes play a pivotal role in the process of fracture healing, particularly during the early phase. However, the specific functions of their cellular subsets and the alterations that occur in the context of osteoporotic fracture (OPF) remain poorly elucidated.

**Methods:**

An OPF model was established using ovariectomized (OVX) C57BL/6 J mice, with sham-operated mice serving as controls. Subsequent single-cell RNA sequencing (scRNA-seq) analysis was performed to identify and characterize monocyte populations within the early fracture microenvironment.

**Results:**

scRNA-seq analysis revealed that monocytes could be classified into four distinct subsets. Monocytes-1 and monocytes-2 were identified as Ly6C^+^ monocytes; specifically, monocytes-1 differentiated into M1 macrophages to mediate inflammatory responses, whereas monocytes-2 proliferated and served as a cellular reservoir to support the differentiation of monocytes-1. Monocytes-3 and monocytes-4 exhibited the potential to differentiate into M2 macrophages. Functionally, monocytes-3 primarily constrained the magnitude of inflammatory responses, while monocytes-4 were involved in orchestrating tissue repair processes, such as angiogenesis. In the local bone tissues of OVX mice at the early fracture stage, all monocyte subsets exhibited a tendency toward reduced abundance.

**Conclusion:**

This study reveals a high degree of functional heterogeneity among monocyte subsets during early fracture healing. The reduction in monocyte subsets in OVX mice impairs the critical pathological transition from inflammatory responses to tissue repair. This impairment may constitute a partial mechanistic basis for the delayed and compromised healing observed in OPF.

## 1 Introduction

Osteoporosis is a systemic skeletal disorder characterized by low bone mass and disrupted bone microstructure. It increases bone fragility and heightens the susceptibility of bones to fractures. It is featured by high morbidity and disability rates, along with poor healing outcomes (1). With the aging of the global population, osteoporotic fracture (OPF) has gradually evolved into a challenging public health concern worldwide. According to the most recent Global Burden of Disease (GBD) analysis, an estimated 178 million new fractures occur worldwide each year, a substantial proportion of which are directly attributable to reduced bone density (2).

Fracture healing is a sequential process consisting of distinct phases of inflammation, repair, and remodeling (3). The early inflammatory response is critical for normal fracture healing (4). Any perturbation that disrupts the homeostasis of the inflammatory phase may lead to delayed fracture healing, as observed after treatment with anti-inflammatory agents such as cyclooxygenase-2 inhibitors (5). Compared with ordinary fractures, the pathophysiological process of OPF healing exhibits distinct abnormal characteristics, including dysregulated and prolonged inflammatory responses, impaired angiogenesis, attenuated osteogenic differentiation, delayed callus mineralization, and adipogenic infiltration of the bone marrow microenvironment. Collectively, these complex pathological alterations can impair bone regeneration.

Monocytes are derived from the bone marrow and distributed in the peripheral circulation (6). In the early stage of fracture healing, monocytes extravasate into the inflammatory locus and differentiate into tissue-resident macrophages and dendritic cells, thereby participating in the inflammatory process indirectly (7). Additionally, upon stimulation with macrophage colony-stimulating factor and receptor activator of nuclear factor-κB ligand (RANKL), monocytes can differentiate into osteoclasts, which in turn contribute directly to bone remodeling (8). Furthermore, monocytes can regulate angiogenesis during the early phase of fracture healing (9).

Traditionally, murine monocytes were broadly classified into two major subsets: classical (Ly6C^+^) and non-classical (Ly6C^−^) monocytes (10). However, recent single-cell transcriptomic breakthroughs in the orthopedic field have fundamentally challenged this binary classification, revealing a highly granular heterogeneity within the monocyte pool during bone regeneration (11). Emerging studies have identified transient, highly specialized monocyte sub-clusters within the early fracture microenvironment that exert distinct functions beyond simple phagocytosis, such as unique subsets dedicated to coupling angiogenesis, driving extracellular matrix remodeling, or orchestrating early crosstalk with skeletal stem cells (9, 12). While this intricate monocyte landscape has been increasingly mapped in normal fracture healing, its spatiotemporal evolution within the compromised microenvironment of OPF remains completely unexplored. In the present study, monocytes were isolated from the local fracture tissues of normal mice and ovariectomized (OVX) mice on days 1, 3, and 7 post-fracture. Dynamic changes in the single-cell transcriptomic profiles of these monocytes were analyzed by single-cell RNA sequencing (scRNA-seq) technology. These monocytes could be categorized into four distinct subsets with heterogeneous functions and states. We further investigated the alterations of these monocyte subsets within the local tissues during the early stage of fracture healing in OVX mice. This study will provide potential therapeutic targets for the development of novel immunomodulatory strategies to facilitate OPF healing.

## 2 Methods

### 2.1 Study design

Eight-week-old female C57BL/6 J mice were randomly assigned to either the ovariectomized (OVX) group or the sham group, undergoing ovariectomy or sham surgery, respectively. At 4 weeks post-surgery, tissue samples were collected to verify the successful establishment of the model. Subsequently, a left mid-tibial fracture model was created in these mice. Local fracture tissues were harvested on day 1, 3, and 7 after fracture, along with tibial tissues from non-fractured mice, for single-cell RNA sequencing and data analysis. All animal experimental protocols used in this study were approved by the Animal Experiment Ethics Committee of Shanghai University of Traditional Chinese Medicine (PZSHUTCM2311300001).

### 2.2 Establishment of the OVX mouse model

Bilateral ovariectomy was performed to establish the mouse model of ovarian hormone deficiency. Mice were anesthetized via inhalation of a 4% isoflurane-oxygen mixture delivered by an anesthesia machine. Upon reaching a surgical plane of anesthesia, the isoflurane concentration was maintained at 2% for the duration of the procedure. The anesthetized mouse was placed in a prone position, and the surgical site (from the lower rib margin to the posterior iliac crest) was shaved and prepared for aseptic surgery. A longitudinal skin incision of approximately 1 cm in length was made along the spinal column, starting just below the posterior iliac crest. The subcutaneous tissue and muscle layer were bluntly dissected. At the muscle layer near the iliac crest on each side, a small incision was made to access the peritoneal cavity. The ovarian pedicle (including blood vessels and fallopian tube) was ligated, and the ovary was subsequently excised. The pedicle stump was returned into the abdominal cavity, and the muscle layer and skin were then sutured closed. For mice in the sham-operated group, a similar procedure was performed, but only the periovarian fat pad was removed while leaving the ovaries intact. Following closure, chlortetracycline ointment was applied to the incision site to prevent postoperative infection. The average duration of the surgery was approximately 5 min, and mice typically recovered from anesthesia within 10 min postoperatively.

### 2.3 Establishment of the left tibial fracture model in mice

Eight weeks following the establishment of the OVX model, a closed tibial fracture was surgically induced in the left hind limb. Mice were anesthetized via inhalation of a 4% isoflurane-oxygen mixture. Upon reaching a surgical plane of anesthesia, the isoflurane concentration was maintained at 2% for the duration of the procedure. The mouse was positioned in lateral recumbency. The fur over the left posterior limb was shaved, and the skin was disinfected with iodine. A longitudinal incision approximately 1 cm in length was made along the anterolateral aspect of the left tibia to expose the bone. The surrounding fascia and muscle were carefully dissected. The skin over the knee joint was retracted proximally to fully expose the joint. A 1-mL syringe needle was inserted into the tibial marrow cavity from the anterolateral aspect of the tibial plateau, advancing approximately 1.5 cm along the long axis of the bone. The needle was then slightly withdrawn, and the tibia was completely osteotomized at the mid-diaphysis using sterile scissors. Following the fracture, the needle was fully re-advanced into the distal fragment to achieve intramedullary fixation. The external portion of the needle hub was cut off, and the skin incision was sutured closed. Chlortetracycline ointment was applied to the incision site postoperatively.

### 2.4 Micro-CT analysis of callus bone mass and trabecular parameters

Tibial specimens preserved in 75% ethanol at 28 days post-fracture were scanned using a micro-CT system. The samples were placed in the scanning chamber, and images were acquired at a voltage of 55 kV, a current of 72 μA, an exposure time of 300 ms per frame, and an isotropic voxel size of 10 μm. Three-dimensional reconstructions were generated from the obtained image sets. The callus region was analyzed using the system’s evaluation software (e.g., Scanco Medical software V6.6) to quantify bone mass parameters, including Bone mineral density (BMD) and Bone volume fraction (BV/TV). Trabecular microarchitecture parameters were also assessed, including Trabecular number (Tb.N), trabecular thickness Trabecular thickness (Tb.Th), and Trabecular separation (Tb.Sp).

### 2.5 Biomechanical property assessment

Tibial specimens harvested at 28 days post-fracture were rehydrated in saline. Biomechanical testing was performed using a mechanical testing system (ElectroForce 3,200). A three-point bending test was conducted by positioning the tibia on two supports. The distance between the two supporting points (a) was measured, and the distances from the loading point to each support (b1 and b2) were recorded. A compressive force was applied to the mid-diaphysis at a constant displacement rate until fracture occurred. Real-time load and displacement data were recorded to generate a load–displacement curve. The force at the point of fracture was recorded as the maximum load (n, in Newtons), reflecting the bone’s structural load-bearing capacity. The stiffness (slope, k) was determined from the linear region of the load–displacement curve. Subsequently, the Elastic Modulus (E), representing the intrinsic stiffness of the healing bone, was calculated using the standard beam theory equation (E = k × a^3/48 × I), where I represents the moment of inertia derived from the cross-sectional geometry. The cortical thickness at the fracture site and the maximum diameter of the fractured cross-section were measured to calculate the cross-sectional area (S) and the moment of inertia (I). A standardized maximum load was subsequently calculated using the formula: Standardized Maximum Load = n × b1 × b2/(S × a).

### 2.6 Histological analysis via hematoxylin and eosin staining

Tibial callus samples were fixed in 4% paraformaldehyde for 48 h at 4 °C, followed by decalcification in 10% ethylenediaminetetraacetic acid (pH 7.4) for 4 weeks. The tissues were then dehydrated through a graded ethanol series, cleared in xylene, and embedded in paraffin. Sections of 5 μm thickness were cut using a microtome and mounted on glass slides. For staining, the sections were deparaffinized in xylene and rehydrated through a descending ethanol series to distilled water. They were then stained with Mayer’s hematoxylin for 8 min to visualize nuclei, followed by a brief rinse in running tap water. Differentiation was performed in 1% acid alcohol for a few seconds, and the sections were blued in Scott’s tap water substitute. Subsequently, the cytoplasm was counterstained with eosin Y solution for 2 min. After staining, the sections were dehydrated through an ascending ethanol series, cleared in xylene, and mounted with a resinous medium. The stained sections were examined under a light microscope to assess general tissue morphology.

### 2.7 Tissue dissociation and single-cell suspension preparation

Fresh mouse tissue samples were obtained and processed immediately under approved institutional review board protocols. Briefly, the tissue was minced into approximately 1–2 mm^3^ fragments using sterile surgical scissors in a petri dish containing ice-cold phosphate-buffered saline (PBS) supplemented with 2% fetal bovine serum (FBS). The fragments were then transferred into a gentle tissue dissociation cocktail, typically consisting of collagenase IV, dispase, and DNase I in PBS, and incubated at 37 °C for 20–45 min with periodic mechanical agitation. The exact enzymatic composition and incubation time were optimized for each specific tissue type.

Following digestion, the cell suspension was sequentially filtered through 70 μm and 40 μm cell strainers to remove undigested fragments and cell aggregates. The filtrate was centrifuged at 300 × g for 5 min at 4 °C. The pellet was resuspended in red blood cell lysis buffer and washed twice with PBS containing 2% FBS. Finally, the cells were resuspended in PBS with 0.04% bovine serum albumin for downstream applications. Cell concentration and viability were determined using an automated cell counter with Trypan Blue exclusion, and only suspensions with viability >80% were used for subsequent scRNA-seq analysis.

### 2.8 GEM generation and 3′ gene expression library construction

Single-cell/nuclei suspensions were loaded to 10x Chromium according to the manufacturer’ s instructions of 10x Genomics Chromium Single-Cell 3′ kit (V3). The following cDNA amplification and library construction steps were performed according to the standard protocol. Libraries were sequenced on an Illumina NovaSeq 6,000 sequencing system (paired-end multiplexing run,150 bp) by Shanghai Personal Biotechnology (Shanghai, China).

### 2.9 Single cell RNA-sequencing and analysis

Raw sequence reads in FASTQ format were processed and aligned to the GRCm39 mouse reference transcriptome using the Cell ranger v7.1.0 pipeline^1^ with default parameters. The resulting gene expression matrices merged together using Seurat package v5. The pre-processing followed the guidelines provided by Seurat V5 tutorial. In short, entries with fewer than 400 genes and greater than 7,500 total genes were filtered to remove empty droplets and probable doublets, respectively, and cells that have >20% mitochondrial counts were also filtered to remove low quality cells. To account for differences in sequencing depth across samples, we normalized expression values for total unique molecular identifiers (UMIs) per cell and log transformed the counts using Seurat Normalize Data function.

### 2.10 Clustering and identification of cell types

For cell clustering, normalized and scaled data were utilized to identify highly variable features using the “Find Variable Features” function (*n* features = 2000). Dimensionality reduction was performed using these features, followed by cell clustering. The resulting cell clusters were visualized using the uniform manifold approximation and projection (UMAP) method and annotated by examining the expression of known marker genes. For each cell type, we rerun the Seurat cluster workflow to identify cell sub-types.

### 2.11 Differential expression and functional enrichment analysis

To identify differentially expressed genes (DEGs), we used the Seurat FindMarkers function based on Wilcox likelihood-ratio test with default parameters, and selected the genes expressed in more than 25% of the cells in a cluster and with an average log(Fold Change) value greater than 1 as DEGs. To investigate the potential functions of DEGs, the gene ontology (GO) and kyoto encyclopedia of genes and genomes (KEGG) analysis were used with the “clusterProfiler” R package. Pathways with p_adj value less than 0.05 were considered as significantly enriched.

### 2.12 Pseudotime analysis

After inputting the gene count matrix, a new dataset for the Monocle object was created, and the functions “reduce Dimension” and “order Cells” were executed to generate a cell development trajectory based on pseudotime. Subsequently, differentially expressed genes between different clusters within each cell type were calculated using the “differential Gene Test” function in Monocle. The “BEAM” function was then utilized to calculate differentially expressed genes at branch points in the trajectory.

### 2.13 Cell–cell communication analysis

The cell–cell interactions between different cell types were evaluated using CellChat (Version 2.1, R package). CellChat takes gene expression data as user input to model the probability of cell–cell communication by integrating gene expression with the existing database consisting of known interaction between signaling ligands, receptors, and their cofactors. In this paper, cell–cell interactions were analyzed individually for different conditions following the default pipeline. Normalized counts data from each condition were used to create CellChat object and applied the recommended pre-processing functions for the analysis of individual datasets with default parameters. CellChatDB was used as the database for inferring cell–cell communication. All categories of ligand–receptor interactions in the database were used in the analysis. Communications involving less than 10 cells were excluded.

### 2.14 Statistical analysis

All data were expressed as the mean ± SD. After homogeneity test of variance, unpaired Student’s *t* test (two-tailed), or repeated ANOVA with Tukey’s *post hoc* tests, or one-way ANOVA with Tukey’s post hoc tests, or two-way ANOVA with Bonferroni post hoc tests were separately used in different experiments as indicated in the figure legend. Prism 6.01 (GraphPad Software Inc.) was used for statistical analysis and *p* < 0.05 was considered statistically significant.

## 3 Results

### 3.1 Establishment of the animal model

**Figure 1:**
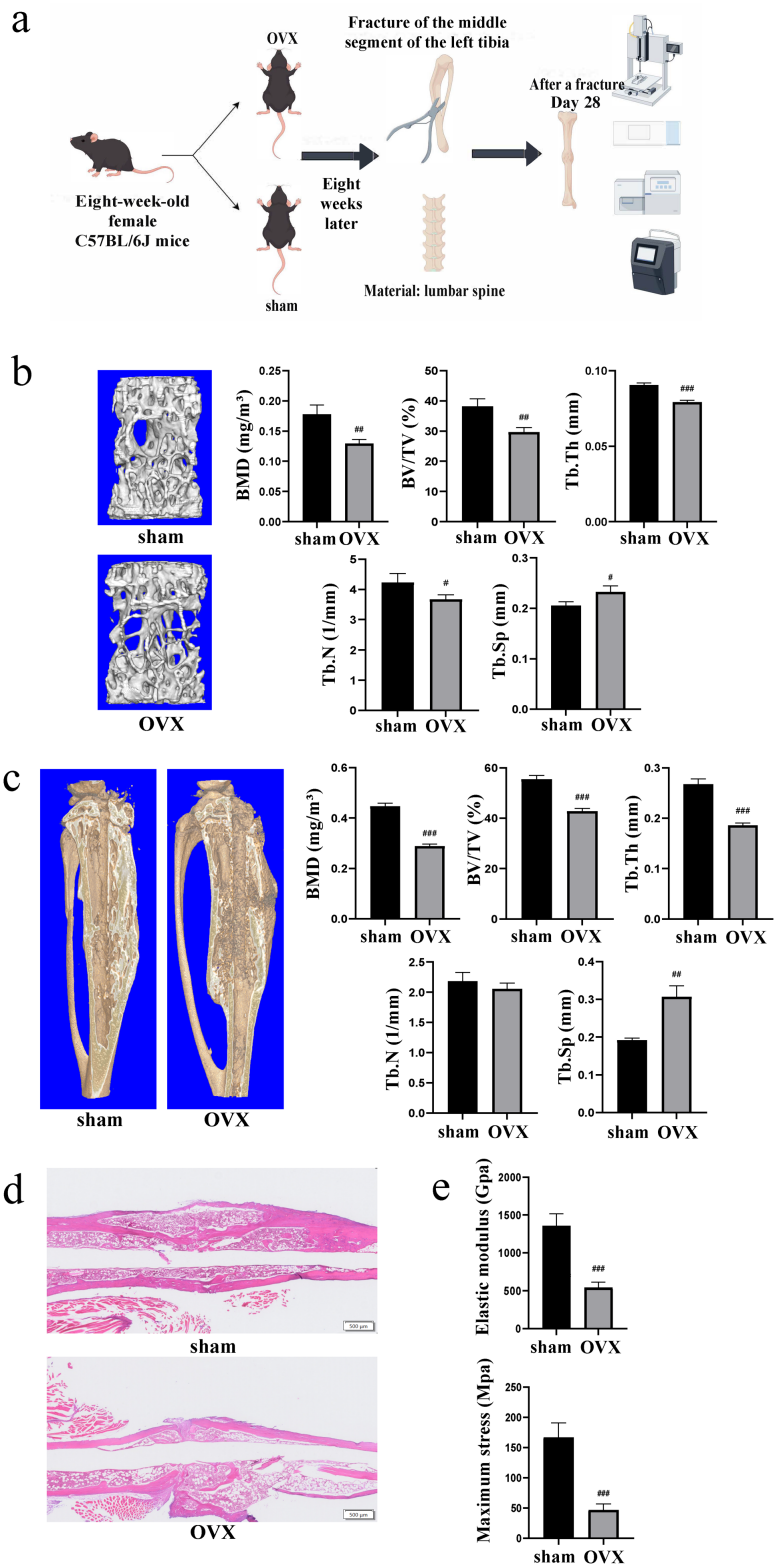
Establishment of the osteoporotic fracture model in mice. **(a)** Schematic diagram of the experimental design, including the ovariectomy or sham surgery, the tibial fracture surgery, and timeline for sample collection. **(b)** Representative Micro-CT images and quantitative analysis of trabecular bone parameters of the lumbar spine at day 28 post-ovariectomy or sham surgery (*n* = 3). **(c)** Representative Micro-CT images and quantitative analysis of the fractured tibia callus 28 days post-fracture (*n* = 3). **(d)** H&E staining images of the callus 28 days post-fracture (scale bar 500 μm). **(e)** Biomechanical testing results (elastic modulus and maximum stress) of the healed tibia at day 28 post-fracture (*n* = 4). Data are presented as mean ± SD, ^#^*p* < 0.05, ^##^*p* < 0.01, ^###^*p* < 0.001. OVX, ovariectomized; Micro-CT, micro-computed tomography; BMD, bone mineral density; BV/TV, bone volume/total volume; Tb.N, trabecular number; Tb.Th, trabecular thickness; Tb.Sp, trabecular separation; M-stress, maximum stress.

The overall workflow is illustrated in Figure 1a. First, 8-week-old female C57BL/6 J mice were subjected to bilateral OVX or sham surgery. On day 28 postoperatively, lumbar vertebrae tissues were harvested from mice in both groups for micro-computed tomography (micro-CT) analysis. Compared with the sham group, the OVX group exhibited a substantial reduction in lumbar bone mineral density (BMD), bone volume fraction (BV/TV), trabecular thickness (Tb.Th) and trabecular number (Tb.N), with a marked increase in trabecular separation (Tb.Sp) (Figure 1b). These findings confirmed the successful establishment of an OVX-induced bone loss mouse model.

Eight weeks after the established OVX mouse model, a midshaft fracture model was constructed in the left tibia of each mouse. Left tibiae were collected for subsequent analyses on day 28 post-fracture. Micro-CT results revealed that relative to the sham group, the OVX group demonstrated marked decreases in BMD, BV/TV and Tb.Th of the healed tibiae, with a downward trend in Tb.N and a prominent increase in Tb.Sp (Figure 1c). Histological analyses demonstrated sparse trabecular bone architecture and an increased number of adipocytes in the bone marrow cavity of the OVX group (Figure 1d). Biomechanical assays indicated that the elastic modulus and maximum stress of the healed tibiae in the OVX group were significantly lower than those in the sham group (Figure 1e). Collectively, healing rate and quality of fractures in OVX mice were markedly inferior to those in sham-operated mice.

### 3.2 Cell clustering analysis of callus tissues

**Figure 2:**
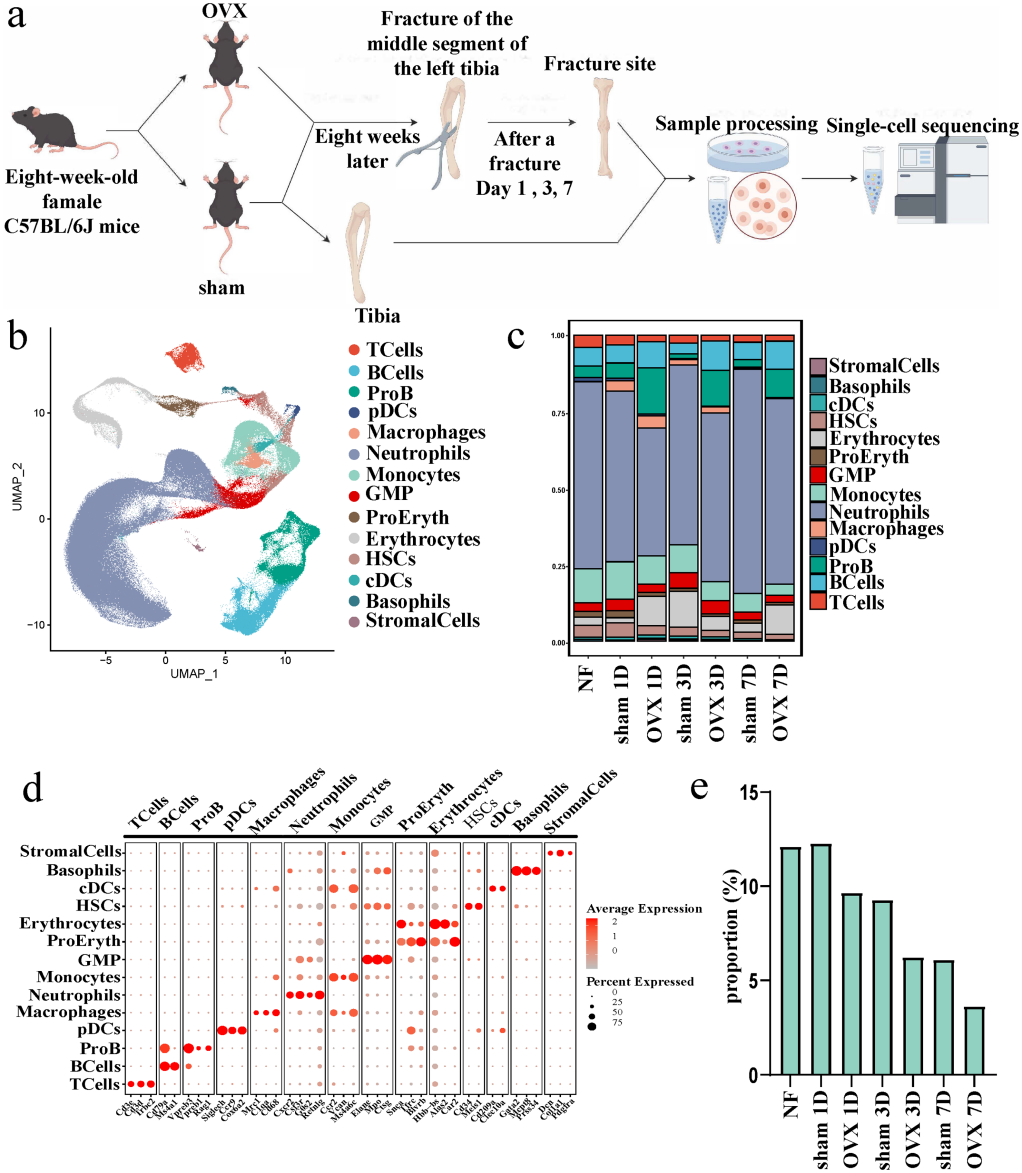
Single-cell transcriptomic landscape of the fracture callus. **(a)** Workflow of the single-cell RNA sequencing experiment. **(b)** UMAP plot visualizing the 14 identified cell clusters in the fracture callus. **(c)** Stacked bar chart showing the proportion of each cell type across different groups and time points. **(d)** Dot plot displaying the expression of top marker genes for each annotated cell cluster. Dot size represents the percentage of cells expressing the gene, and color intensity indicates average expression levels. **(e)** Bar chart quantifying the proportion of monocytes among total cells in the local fracture tissues across the different experimental groups (*n* = 3). OVX, ovariectomized; UMAP, uniform manifold approximation and projection; cDCs, conventional dendritic cells; HSCs, hematopoietic stem cells; ProEryth, proerythroblasts; GMP, granulocyte-macrophage progenitors; pDCs, plasmacytoid dendritic cells; ProB, pro-B cells; NF, non-fractured; 1D, day 1; 3D, day 3; 7D, day 7.

Tibial tissues were harvested from non-fractured (NF), local fracture sites of sham-operated and OVX mice on days 1, 3, and 7 post-fracture, for single-cell sequencing (Figure 2a). Following data integration of the single-cell transcriptomes, dimensionality reduction and clustering analyses were performed. Using differentially expressed and established cell-type marker genes, we identified 14 distinct cell populations, including stromal cells, basophils, conventional dendritic cells, hematopoietic stem cells, erythrocytes, proerythroblasts, granulocyte-macrophage progenitors, monocytes, neutrophils, macrophages, plasmacytoid dendritic cells, pro-B cells, B cells, and T cells. The proportion of each cell population relative to the total cells was calculated (Figures 2b–d).

The percentage of monocytes among total cells in the local fracture tissues of each group is presented in Figure 2e. Compared with the NF group, the proportion of monocytes in the sham and OVX groups exhibited a gradual decline on days 3, and 7 post-fracture. Moreover, at each time point, the proportion of monocytes in the OVX group also tended to be lower than that in the sham group. As a consequence, the quantity and functional status of monocytes during the early stage of fracture healing are impaired under osteoporotic conditions.

### 3.3 Identification and expression characteristics of monocyte subsets

**Figure 3:**
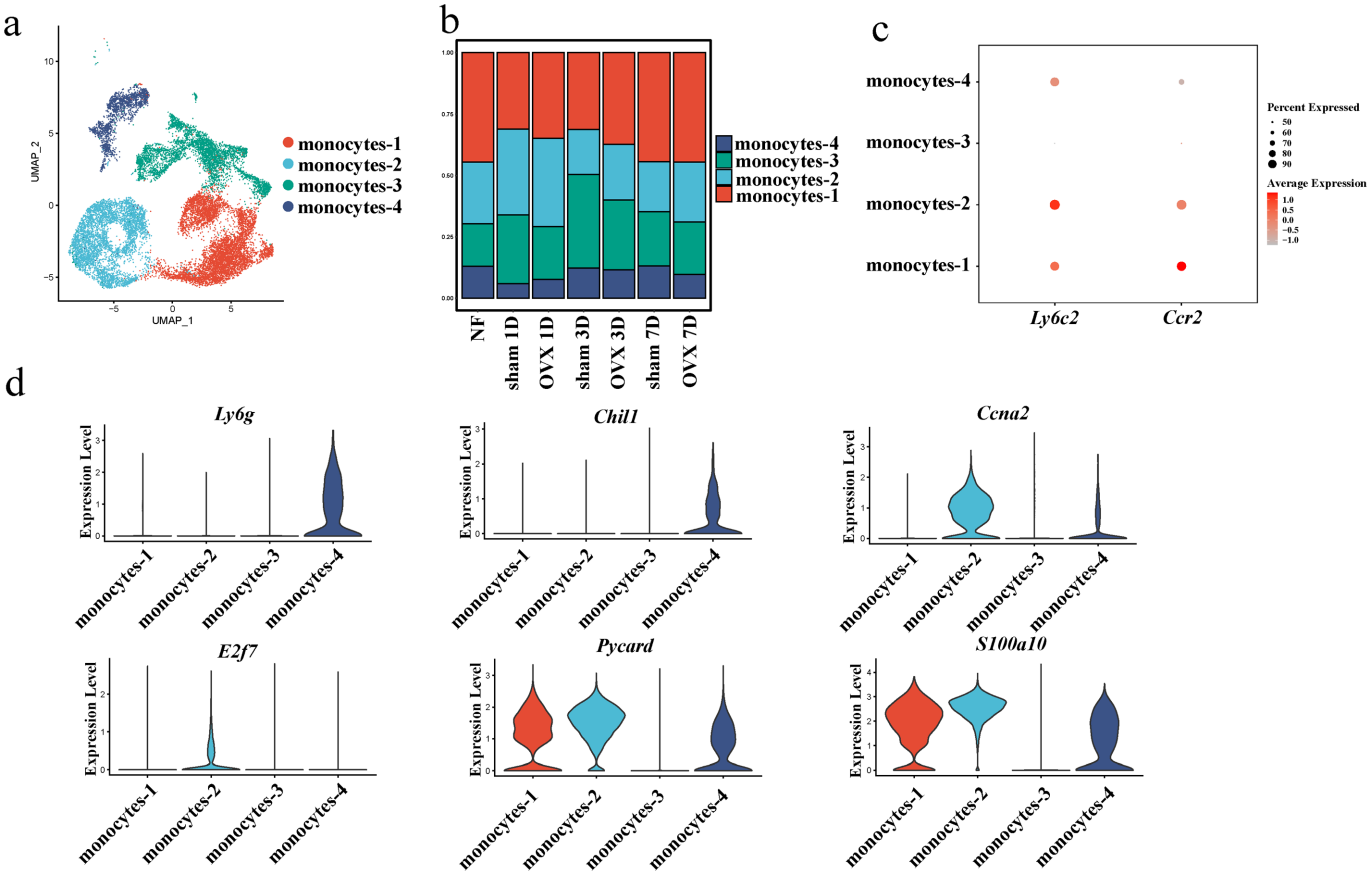
Identification and characterization of monocyte heterogeneity in the early stage of fracture healing. **(a)** UMAP plot showing the re-clustering of monocytes into four distinct subsets. **(b)** Proportions of the monocyte subsets across different groups and time points. **(c)** Dot plot of classical monocyte marker genes (*Ly6c2*, *Ccr2*) across the four subsets. **(d)** Violin plots showing the expression levels of specific marker genes (*Ly6g*, *Chil1*, *Ccna2*, *E2f7*, *Pycard*, *S100a10*). OVX, ovariectomized; UMAP, uniform manifold approximation and projection; NF, non-fractured; 1D, day 1; 3D, day 3; 7D, day 7.

Monocytes were subjected to further analysis. Dimensionality reduction and visualization were performed, which classified monocytes into four distinct subsets (Figure 3a). The proportion of each subset relative to the total monocyte population was presented as stacked bar charts (Figure 3b). Ly6c and chemokine (C-C motif) receptor 2 (Ccr2) are adopted for the classification of monocyte subsets (13). In the present study, monocytes-3 and monocytes-4 exhibited relative low expression levels of *Ly6c* and *Ccr2*, whereas monocytes-1 and monocytes-2 demonstrated high expression of these two genes (Figure 3c). The signature genes specific to each of the four subsets were selected and visualized using violin plots (Figure 3d). Specifically, monocytes-4 displayed marked upregulation of lymphocyte antigen 6 complex, locus G (*Ly6g*) and chitinase-like protein 1 (*Chil1*). Monocytes-2 expressed cyclin A2 (*Ccna2*) and E2F transcription factor 7 (*E2f7*). In addition, PYD and CARD domain containing gene (*Pycard*) and S100 calcium-binding protein A10 (*S100a10*) were substantially expressed in monocytes-1, monocytes-2 and monocytes-4.

**Figure 4:**
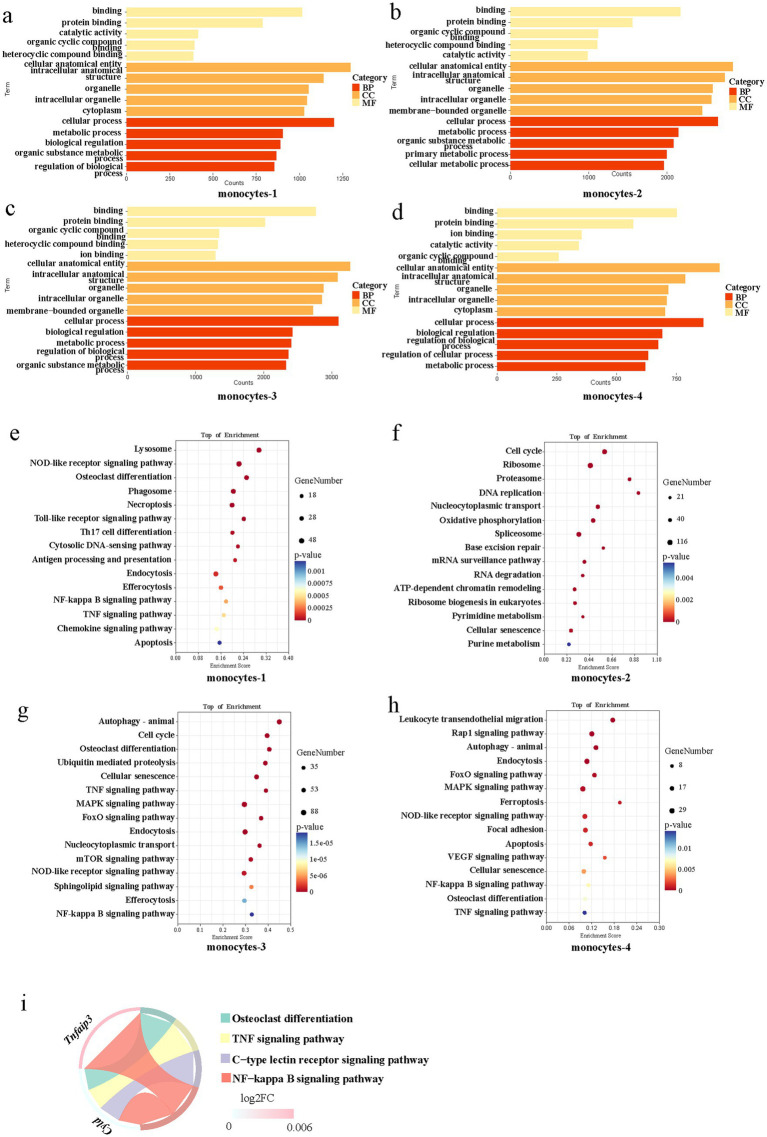
Functional enrichment analysis of the monocyte subtypes in the early stage of fracture healing. **(a–d)** Gene ontology enrichment analysis of upregulated genes for each monocyte subset. **(e–h)** Kyoto encyclopedia of genes and genomes pathway enrichment analysis for each monocyte subset. **(i)** Chord diagram illustrating the linkage between specific differentially expressed genes (*Tnfaip3*, *Cyld*) and enriched signaling pathways. BP, biological process; CC, cellular component; MF, molecular function; NOD, nucleotide-binding oligomerization domain; TNF, tumor necrosis factor; NF-kappa B, nuclear factor kappa-B; ATP, adenosine triphosphate; MAPK, mitogen-activated protein kinase; mTOR, mechanistic target of rapamycin; FoxO, forkhead box O; VEGF, vascular endothelial growth factor.

Gene ontology (GO) functional enrichment analysis on the upregulated genes of each monocyte subset revealed enriched GO terms for all monocyte subsets included binding, cellular process, metabolic process, biological regulation, and others (Figures 4a–d). The results of kyoto encyclopedia of genes and genomes (KEGG) pathway analysis revealed that monocytes-1 were significantly enriched in pathways including lysosome, the nucleotide-binding oligomerization domain (NOD)-like receptor signaling pathway, phagosome, necroptosis, Toll-like receptor signaling pathway, tumor necrosis factor (TNF) signaling pathway, and others. Monocytes-2 were enriched in pathways such as cell cycle, ribosome, proteasome, DNA replication, nucleocytoplasmic transport, oxidative phosphorylation, splicing, and others. In addition to signaling pathways including TNF, mitogen-activated protein kinase, forkhead box O (FoxO), mechanistic target of rapamycin (mTOR), and NOD-like receptor pathways, monocytes-3 also exhibited enrichment in pathways associated with autophagy, cell cycle, cellular senescence, endocytosis, and efferocytosis. Compared with the other subsets, monocytes-4 were enriched in pathways such as leukocyte transendothelial migration, Rap1-signaling pathway, and vascular endothelial growth factor (VEGF)-signaling pathway (Figures 4e–h).

Analysis of monocytes-3 revealed that although pro-inflammatory signaling pathways such as nuclear factor kappa-B (NF-κB) and TNF were upregulated in this subset, the gene expression levels of negative feedback regulators, such as *Tnfaip3* and *Cyld*, within these pathways were also elevated (Figure 4i). Consequently, monocytes-3 exert a bidirectional regulatory effect on inflammation-associated signaling pathways.

**Figure 5:**
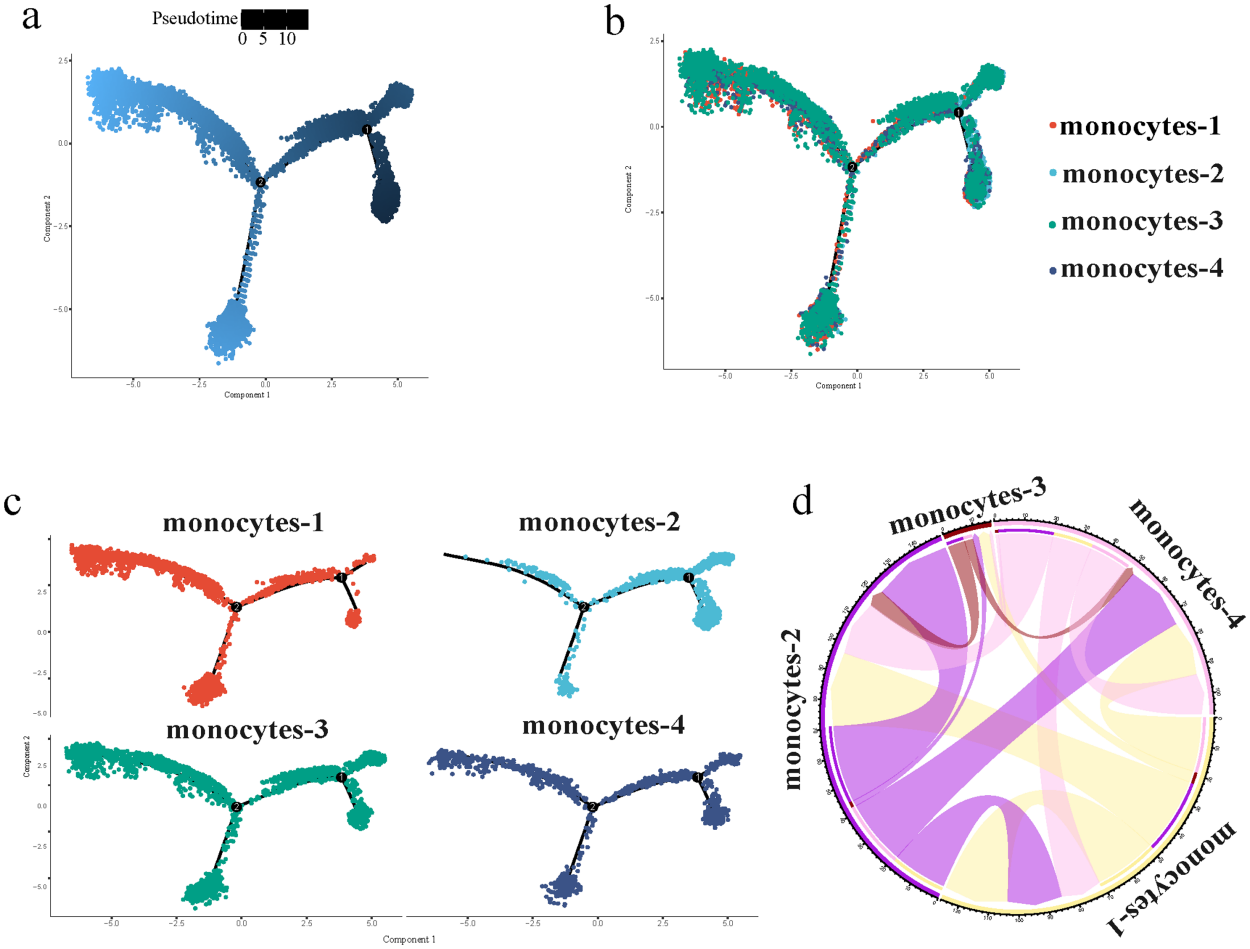
Pseudotime trajectory and interaction analysis of monocyte subsets in the early stage of fracture healing. **(a)** Pseudotime trajectory axis generated by Monocle 2 analysis. **(b)** Distribution of the monocyte subsets along the pseudotime trajectory. **(c)** Split-view of the trajectory for each subset. **(d)** Circos plot visualizing the relationships or potential transitions between the four monocyte subsets.

We performed pseudotime trajectory analysis on the monocyte subsets (Figures 5a–c). Monocytes-1 were concentrated in the middle and terminal segments of the pseudotime axis, monocytes-2 were clustered at the initial segment, while monocytes-3 and monocytes-4 were distributed across all the segments. We further conducted intercellular communication analysis on scRNA-seq data using the CellChat tool and visualized the signal intensity among different subsets by chord diagrams (Figure 5d). Among all monocyte subsets, stronger intercellular communication was observed between monocytes-1, monocytes-2 and monocytes-4.

### 3.4 Differences in monocyte subsets between sham and OVX groups

**Figure 6:**
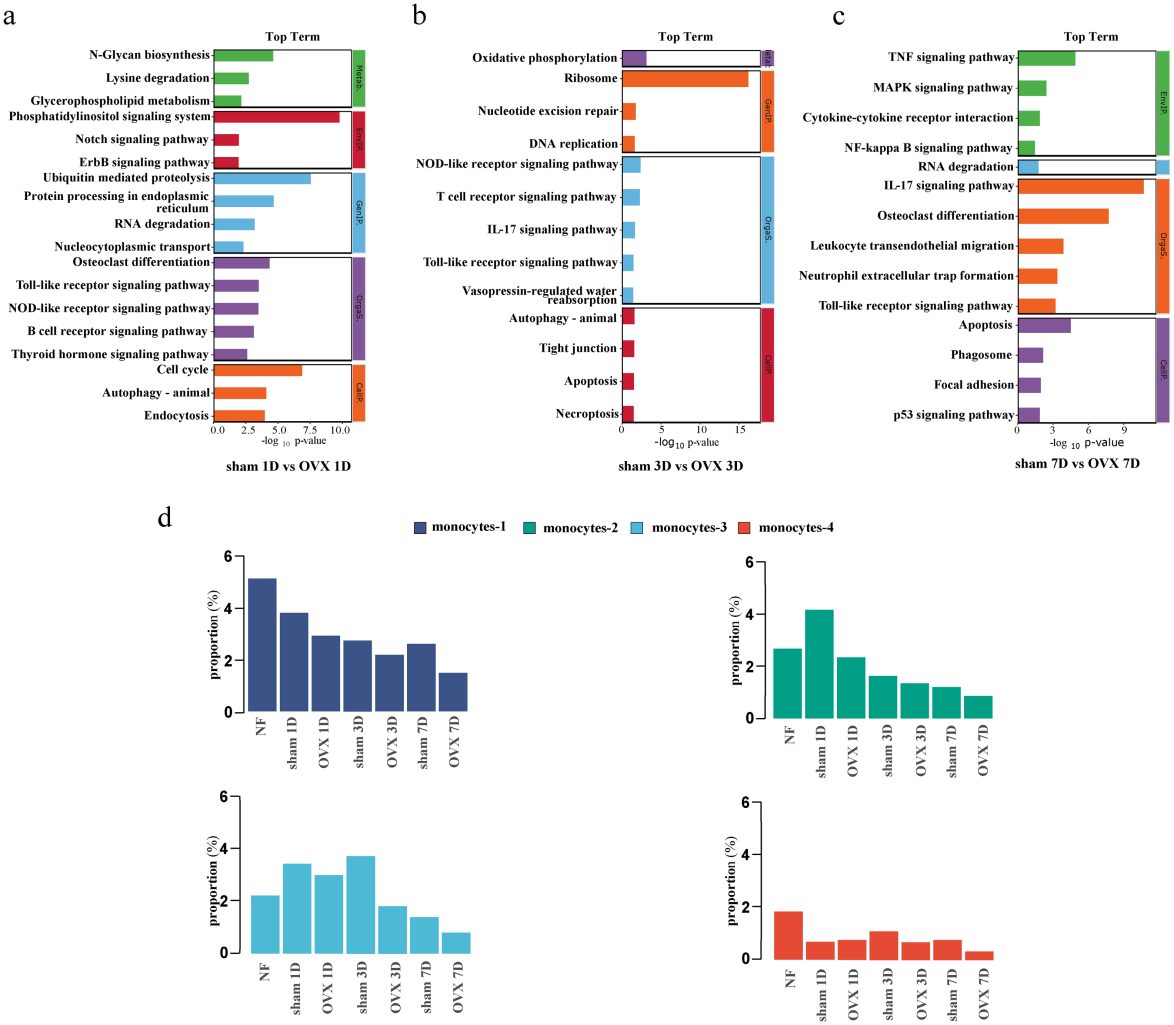
Pathway enrichment analysis and dynamic changes in monocyte subset proportions among different groups in the early stage of fracture healing. **(a–c)** Bar charts showing the top enriched pathways of downregulated genes in the OVX group compared with those in the sham group at days 1, 3, and 7 post-fracture. **(d)** Bar chart illustrating the proportion of monocyte subsets across different groups at days 1, 3, and 7 post-fracture. OVX, ovariectomized; NF, non-fractured; 1D, day 1; 3D, day 3; 7D, day 7.

We performed enrichment analysis on the downregulated genes of monocytes in the OVX group relative to the sham group at each time point (Figures 6a–c). On day 1 post-fracture, enriched pathways included N-glycan biosynthesis, lysine degradation, ubiquitin-mediated proteolysis, and phosphatidylinositol signaling system. On day 3, enriched pathways were oxidative phosphorylation, ribosome, nucleotide excision repair, and DNA replication. On day 7, enriched pathways were the TNF signaling pathway, IL-17 signaling pathway, osteoclast differentiation, and apoptosis.

Differences in the proportion of each monocyte subset relative to the total cell population across all the groups were also analyzed (Figure 6d). Compared with the NF group, the proportion of monocytes-1 in the local fracture tissues of sham-operated mice manifested a continuous decreasing trend on days 1, 3, and 7 post-fracture. The proportions of monocytes-2 and monocytes-3 exhibited an upward trend on day 1 post-fracture. Thereafter, the proportion of monocytes-2 gradually decreased, while that of monocytes-3 continued to rise until day 3 and declined on day 7. The proportion of monocytes-4 decreased on day 1 post-fracture and demonstrated a slight increasing trend from day 3 onward. Compared with the sham group, the proportions of monocytes-1, monocytes-2, and monocytes-3 in the local fracture tissues of OVX mice exhibited a decreasing trend at all-time points post-fracture, whereas the proportion of monocytes-4 decreased on days 3 and 7 post-fracture.

## 4 Discussion

Monocytes exhibit high plasticity and heterogeneity. They can undergo cross-differentiation into distinct subsets in response to environmental changes. Based on the differences in the surface markers, murine monocytes were classified into two subsets: Ly6C^+^ and Ly6C^−^ monocytes, previously (14).

Murine Ly6C^+^ monocytes possess robust antimicrobial and phagocytic capacities. They are selectively recruited to injured tissues and lymph nodes *in vivo* and produce high levels of TNF-*α* and IL-1 during infection or tissue injury (15, 16). Consequently, Ly6C^+^ monocytes are designated as inflammatory monocytes. Additionally, these cells can differentiate into M1 macrophages (17). Under homeostatic conditions, Ly6C^+^ monocytes can also differentiate into Ly6C^−^ monocytes in the circulation and remain confined within the vasculature. Via the chemokine receptor CX3CR1, Ly6C^−^ monocytes bind to endothelial cells through the lymphocyte function-associated antigen 1/intracellular cell adhesion molecule 1 (LFA-1/ICAM1) axis to patrol the luminal side of small-vessel endothelium. In vascular inflammation, Ly6C^−^ monocytes can be recruited to local tissues initially. They may differentiate into M2 macrophages and secrete anti-inflammatory cytokines later, thereby contributing to tissue repair (18).

Building upon two canonical subsets (Ly6C^+^ and Ly6C^−^ monocytes), four additional distinct monocyte subsets were identified. Ly6C^+^ monocytes encompass monocytes-1 and monocytes-2. Ly6C^−^ monocytes include monocytes-3 and monocytes-4.

belong to the Ly6C^+^ monocyte subset. In addition to the high expression of *Ly6c2* and *Ccr2*, monocytes-1 and monocytes-2 also exhibit elevated expression of *Pycard*, *S100a10*, and other genes. *Pycard* and *S100a10* mediate the recruitment of Ly6C^+^ monocytes (19). Specifically, *Pycard* can enhance inflammasome activity (20). Meanwhile, the enriched terms of monocytes-1 include lysosome, phagocytosis, necrosis, antigen processing, apoptosis, endocytosis, chemokines, as well as multiple inflammation-associated signaling pathways. Consequently, monocytes-1 may represent the primary subset of Ly6C^+^ monocytes involved in mediating inflammatory responses. Given that Ly6C^+^ monocytes can mature into M1 macrophages, monocytes-1 are responsible for executing the differentiation program toward M1 macrophages among the monocyte subsets.

Monocytes-2 express *Ccna2* and *E2f7*. CCNA2 is a core molecule regulating the cell cycle, which drives the transitions between G1/S and G2/M phases to ensure the orderly progression of DNA replication and mitosis (21). In contrast, E2F7 binds to the promoters of G1/S phase genes such as *Ccna2* to arrest the S phase progression (22). The high co-expression of *Ccna2* and *E2f7* suggests the existence of bidirectional regulation of cell proliferation within monocytes-2. The enriched terms of genes expressed in monocytes-2 are also involved in processes related to the central dogma, ranging from DNA replication, RNA transcription, to protein synthesis, as well as the energy supply and regulatory mechanisms supporting these processes. Combined with the upward trend in the proportion of monocytes-2 on day 1 post-fracture, monocytes-2 recruited to the fracture site at the initial stage of fracture may undergo robust cell proliferation. Previous study has demonstrated that classical Ly6C^+^ monocytes proliferate locally in a colony-stimulating factor 1 receptor-dependent manner before differentiating into lung interstitial macrophages (23), which further supports our findings. Pseudotime trajectory analysis revealed that monocytes-2 were located at the initial stage of the pseudotime axis, while monocytes-1 were distributed in the middle and terminal stages. This suggests that monocytes-2 have the potential to differentiate into the monocytes-1 state, thereby serving as a cellular reservoir to replenish monocytes-1. The differentiation and transition from proliferating monocytes-2 to the mature inflammatory monocytes-1 state may be orchestrated by specific microenvironmental cues. For instance, signaling axes such as macrophage colony-stimulating factor and its receptor, as well as local chemokines like C-C motif chemokine ligand 2 interacting with CCR2, could drive this lineage progression and functional maturation (24). Further experimental validation is needed to fully elucidate the exact molecular triggers of this transition.

After a fracture, monocytes-1 are recruited to the local fracture site and differentiate into M1 macrophages to participate in the inflammatory response. It leads to a decreasing trend in the proportion of monocytes-1 on day 1 post-fracture. Meanwhile, monocytes-2 proliferate rapidly with an increased cell proportion, and replenish monocytes-1, resulting in a decline in their own proportion. As the inflammatory response subsides, decreasing trends in the proportions of monocytes-1 and monocytes-2 are reversed by day 7 post-fracture, which indicated that the cellular transformation process gradually attenuates or terminates at this time point.

Cell communication analysis indicated that there is a close correlation among monocytes-1, monocytes-2, and monocytes-4, whereas monocytes-3 exhibits minimal interactions with the other three subsets. Combined with pseudotime analysis, monocytes-3 presents a relatively uniform distribution along the entire pseudotime axis, with low expression levels of multiple markers, including *Ly6c2*, *Ccr2*, *Ly6g*, *Chil1*, *Ccna2*, and *Pycard*. Therefore, monocytes-3 may represent a relatively stable subset among monocytes. In addition to common inflammation-related signaling pathways, significantly enriched terms in monocytes-3 also include processes such as autophagy, efferocytosis, and endocytosis. These processes can collectively function to clear damage-associated molecular patterns in the injured microenvironment, inhibit excessive inflammation, and drive the inflammation-repair transition (25). Although the NF-κB signaling pathway is activated, the expression of its negative feedback regulators (for example, *Tnfaip3*, *Cyld*) is simultaneously upregulated (26), suggesting a dual regulation of inflammation-related pathways by monocytes-3. As monocytes-3 belong to Ly6C^−^ monocytes, they can also differentiate into M2 macrophages and promote tissue repair. Collectively, monocytes-3 may serve as the major monocyte subset involved in regulating the intensity of inflammatory responses and driving the inflammation-repair transition.

Monocytes-4, which also belong to Ly6C^−^ monocytes, express multiple markers such as *Ly6g*, *Chil1*, *Pycard*, and *S100a10*. They exhibit close interactions with monocytes-1 and monocytes-2, indicating that monocytes-4 display active cellular functions. The enriched pathways of upregulated genes in monocytes-4 include leukocyte transendothelial migration, focal adhesion, and the VEGF signaling pathway. Its highly expressed *S100a10* also promotes the chemotaxis of monocytes to inflammatory sites. It can simultaneously provide new blood vessels for tissues in the repair stage by enhancing the proliferation, migration, and lumen formation of endothelial cells. This suggests that monocytes-4 may play a role in monocyte migration, infiltration, and angiogenesis promotion in the early stage of fracture. Monocytes can secrete large vesicles rich in transforming growth factor-β3 (TGF-β3), VEGF-A, and C-X-C motif chemokine ligand 12 (CXCL)-12 to act on capillary formation areas, exerting a pro-angiogenic function. They can also recruit and migrate more monocytes through CXCL-12-mediated chemotaxis to rapidly form a capillary network.

Notably, *Ly6g*, an upregulated gene of monocytes-4, is not only a specific surface marker of neutrophils but also expressed in activated macrophages. In addition to regulating the release of pro-inflammatory factors, it is also involved in modulating cellular adhesion and migration functions (27). Monocytes-4 also highly express *Chil1*, a characteristic gene typically expressed in M2-type macrophages. In a lung injury study, a subset of atypical macrophages with high *Ly6g* expression was identified, which exerts a regulatory role in alveolar epithelial regeneration (28). Accordingly, monocytes-4 can migrate and infiltrate into the local injury site after fracture, and promote early tissue repair through pathways such as angiogenesis promotion.

In mice, Ly6C^−^ monocytes are generally derived from Ly6C^+^ monocytes via an nuclear receptor subfamily 4 group A member 1 (NR4A1)-dependent pathway (29). This process of Ly6C^+^ monocytes differentiating into Ly6C^−^ monocytes or migrating from the circulatory system to tissues is rapid (30). After a fracture, the proportion of monocytes-3 in the local injured bone tissue increases rapidly. Monocytes-3 limit and balance the extent of inflammatory responses during the early inflammatory phase post-injury. In contrast, the proportion of monocytes-4 decreases. Under homeostatic conditions, monocytes-4 are continuously derived from the conversion of Ly6C^+^ monocytes. However, under the pathological condition of fracture, a large number of Ly6C^+^ monocytes differentiate into M1 macrophages to perform inflammatory functions. As a result source of monocytes-4 is reduced. As the inflammatory response gradually weakens, the proportion of monocytes-3 decreases accordingly. Meanwhile, the source of some monocytes-4 is gradually restored, and they differentiate into M2-type macrophages. Consequently, a slight upward trend in the proportion of monocytes-4 is observed compared with that on day 1 post-fracture.

The present study also found that at different time points in the early stage of fracture, the proportion of total monocytes in the callus tissue of OVX mice presented a decreasing trend compared with that of normal fractured mice. In addition, temporal changes in the proportions of various monocyte subsets in the local bone tissue during the early fracture healing phase of OVX mice were similar to those in the sham group. However, overall proportions were lower than those in the sham group at the corresponding time points.

Under physiological conditions, estrogen exerts an anti-inflammatory effect mainly by inhibiting the NF-κB pathway (31). It downregulates the transcription of monocyte chemoattractant protein-1 in stromal cells and endothelial cells. It also controls the migration of circulating Ly6C + monocytes into tissues via the CCR2 receptor (32). Previous study suggested that an imbalance in monocyte subsets is a characteristic feature of postmenopausal osteoporosis (11). This study revealed that on day 1 post-fracture, the proportions of monocytes-2 and monocytes-3 in the sham group demonstrated a marked upward trend, whereas OVX mice lost this phenotype. This suggests that under the pathological condition of fracture, the mobilization capacity of monocytes-2 and monocytes-3 during the acute inflammatory phase are impaired. Previous studies have also found that on day 1 post-fracture in mice, there was a peak expression of M1 macrophages, M2 macrophages, and inflammatory factors (33). These indicators markedly decrease in OVX mice, suggesting a reduction in the intensity of the inflammatory response in local bone tissue during the acute phase of fracture in the absence of estrogen. This is consistent with the results of this study. Additionally, on days 3 and 7 post-fracture, the proportions of monocytes-3 and monocytes-4 in OVX mice demonstrated a decreasing trend compared with those in the sham group. Monocytes-3 and monocytes-4 are Ly6C^−^monocytes that can differentiate into M2 macrophages. The reduction in monocytes-3 and monocytes-4 may lead to prolonged inflammatory responses and delayed or weakened injury repair processes after fracture. Consistent with the present findings, deletion of estrogen receptor *α* in macrophages led to impaired phagocytic capacity and reduced expression levels of IL-4-induced M2 macrophage markers such as Ppard, Pparg, Chi3l3, Retnla, and Tgfb1. These markers are closely associated with the anti-inflammatory and tissue repair functions of M2 macrophages (34, 35). Taken together, estrogen deficiency severely impair the initial mobilization and local expansion of the Ly6C^+^ monocyte pool (monocytes-2) within the early fracture microenvironment, and this may disrupts the subsequent generation of regulatory and reparative Ly6C^−^ subsets (monocytes-3 and monocytes-4). Ultimately, this estrogen deficiency-driven imbalance in monocyte subpopulations prolongs the local inflammatory phase and restricts subsequent tissue repair.

While this study offers novel cellular and mechanistic insights into monocyte heterogeneity during OPF healing, several limitations should be acknowledged. First, this research relies exclusively on a murine OVX model. Although it effectively mimics postmenopausal osteoporosis, inherent interspecies differences between mice and humans remain with regard to bone microarchitecture and immune responses (36). While procuring early-stage human fracture callus tissue poses substantial ethical and practical challenges, validating our findings in clinical cohorts remains a critical step toward establishing clinical translatability. Second, due to temporal and resource constraints, the sample size for scRNA-seq was limited; further studies are therefore essential to validate the transcriptomic results of this work. Finally, the precise molecular mechanisms underlying intercellular crosstalk and monocyte subset transitions warrant further in-depth investigation.

## 5 Conclusion

This study preliminarily explored the dynamic changes and functional division among different monocyte subsets during the early stage of fracture healing. Monocytes-2 act as a cell expansion pool, providing a cellular source for monocytes-1 and assisting the latter in driving the inflammatory response in the early stage of injury. Monocytes-3 exert a surveillance role to maintain an appropriate intensity of inflammatory response. Monocytes-4 tend to differentiate into anti-inflammatory/repairative M2 macrophages, promote the regeneration of blood vessels and other tissues at the injury site. Under the osteoporotic condition induced by ovariectomy, proportions of all monocyte subsets decrease in the early stage of fracture, thereby affecting various pathological processes from inflammatory response to tissue repair. This may be part of the mechanism underlying the delayed healing process and reduced healing quality of osteoporotic fractures.

## Data Availability

The datasets presented in this study can be found in online repositories. The names of the repository/repositories and accession number(s) can be found at: https://www.ncbi.nlm.nih.gov/, https://www.ncbi.nlm.nih.gov/sra/PRJNA1424248.

## Footnotes

1
https://www.10xgenomics.com/
